# Defining three ferroptosis-based molecular subtypes and developing a prognostic risk model for high-grade serous ovarian cancer

**DOI:** 10.18632/aging.205857

**Published:** 2024-05-24

**Authors:** Xiang Sun, Wenbin He, Baohua Lin, Weiming Huang, Danping Ye

**Affiliations:** 1 Department of Obstetrics and Gynecology, Guangzhou Women and Children’s Medical Center, Guangzhou Medical University, Guangzhou, Guangdong 510623, China; 2 Department of Otolaryngology, Lanzhou University Second Hospital, Gansu 730030, China

**Keywords:** ovarian cancer, ferroptosis, molecular subtype, prognosis, differential expressed genes, immunotherapy

## Abstract

Background: As a newly defined regulated cell death, ferroptosis is a potential biomarker in ovarian cancer (OV). However, its underlying mechanism in tumor microenvironment (TME) and clinical prediction significance in OV remained to be elucidated.

Methods: The transcriptome data of high-grade serous OV from The Cancer Genome Atlas (TCGA) database were downloaded. Molecular subtypes were classified based on ferroptosis-correlated genes from the FerrDb database by performing consensus clustering analysis. The associations between the subtypes and clinicopathologic characteristics, mutation, regulatory pathways and immune landscape were assessed. A ferroptosis-related prognostic model was constructed and verified using International Cancer Genome Consortium (ICGC) cohort and GSE70769.

Results: Three molecular subtypes of OV were defined. Patients in subtype C3 tended to have the most favorable prognosis, while subtype C1 showing more mesenchymal cells, increased immune infiltration of Macrophages_M2, lower tumor purity, and epithelial-to-mesenchymal transition (EMT) features had the poorest prognosis. A ferroptosis-related risk model was constructed using 8 genes (PDP1, FCGBP, EPHA4, GAS1, SLC7A11, BLOC1S1, SPOCK2, and CXCL9) and manifested a strong prediction performance. High-risk patients had enriched EMT pathways, more Macrophages_M2, less plasma cells and CD8 cell infiltration, greater tendency of immune escape and worse prognosis. The risk score has negatively correlated relation with LAG3, TIGIT, CTLA4, IDO1, CD27, ICOS, and IL2RB but positively correlated with PVR, CD276, and CD28. Moreover, low-risk patients were more sensitive to Cisplatin and Gefitinib, Gemcitabine.

Conclusions: Our results could improve the understanding of ferroptosis in OV, providing promising insights for the clinical targeted therapy for the cancer.

## INTRODUCTION

As reported in the most recent Global Cancer Statistics, ovarian cancer (OV) is one of the most frequent causes of female cancer-related mortality [1]. OV is one of the most frequent gynecologic tumors, ranking the third in incidences and deaths [1, 2]. As a predominant type of OV, ovarian epithelial carcinoma accounts for 90% of all OV cases, and serous ovarian carcinoma is the most prevalent subtype [3]. As currently we lack effective screening methods, serous OV is often diagnosed at a late stage [4]. 5-year survival rate of patients with serous OV is 43%, which is noticeably less than that of clear cell carcinoma (66%), mucinous (71%), and endometroid (82%) [3]. Standard therapeutic strategy for newly diagnosed cases is cytoreductive surgery, systematic pelvic and para-aortic lymph node dissection and adjuvant platinum-based chemotherapies for patients with higher stage or higher grade [4]. However, drug resistance and adverse reactions are great challenges in clinical treatment. Though immunotherapies could improve the prognosis of recurrent OV, durable response rate and disease control rate of patients are generally lower than 20% and 50%, respectively [5, 6]. Hence, developing potential therapeutic biomarkers for prognostic prediction and personalized therapies is of great significance.

Cell death is a life process that occurs under physiological or pathological conditions [7]. Ferroptosis is a newly defined regulated cell death resulting from an overload of iron-dependent lipid peroxidation on cellular membranes [8]. Ferroptosis can be induced via oxidative turbulence, which is mainly modulated by system Xc- and glutathione peroxidase 4 [9, 10]. Different from regulated cell death such as apoptosis, autophagy or necroptosis, ferroptosis is manifested as mitochondrial atrophy and reduction of mitochondrial crista [11]. Study showed that ferroptosis is closely involved in cancer development, progression, and suppression [11]. Inducing ferroptosis in tumor cells could be a promising strategy in cancer therapies including radiotherapy (RT), immunotherapy, chemotherapy and targeted therapies [12]. Although ferroptosis-related differential expressed genes (DEGs) have been used to develop a prognostic model for OV [2, 13], prognostic significance of ferroptosis-based genes in OV classification and prognostic prediction and response to anti-tumor therapy remains to be investigated.

This study collected a total of 361 primary high-grade serous OV cases from The Cancer Genome Atlas (TCGA) database. Three heterogeneous subtypes were classified and their relationships with genomic alteration, clinicopathological characteristics, and immune characteristics were evaluated. Moreover, a prognostic risk model was developed and validated to be able to accurately predict OV prognostic outcomes and patients’ responses to chemotherapy/immunotherapy. Collectively, our findings could improve the current understanding underlying the mechanism of ferroptosis in OV, contributing to the development of the clinical therapy for OV.

## MATERIALS AND METHODS

### Data source and pro-processing

We downloaded the RNA-Seq data of a total of 361 primary high-grade serous OV samples (non-Stage I and non-Grade 1) from TCGA (https://portal.gdc.cancer.gov/) database using Genomic Data Commons Application Programming Interface. The data in TCGA-OV cohort were pre-processed to obtain qualified samples with high-grade serous OV and survival time > 30 days and remove those without survival time, survival states or clinical follow-up information. Following that, FPKM was converted to TPM, and ensembl gene IDs were transformed into gene symbol IDs. The median was used to express the genes with various gene symbol IDs.

The gene expression profiles of 85 OV samples from GSE70769 in the Gene-Expression Omnibus (GEO; https://www.ncbi.nlm.nih.gov/geo/) database were collected. Normalized gene expression profiles and clinical information were acquired from the ovarian cancer-Australia (OV-AU) project in ICGC (https://icgc.org) database and 93 OV samples were obtained. The probe matching to multiple genes in GEO were removed. The median was used to express the genes. TCGA-OV served as a training cohort, while GSE70769 and ICGC-AU were used as independent validation cohorts. Subsequently, we collected ferroptosis-related genes from the FerrDb database (http://www.zhounan.org/ferrdb).

### Identification of ferroptosis-based subtypes

Ferroptosis-related genes (with *P* < 0.05) in TCGA-OV were identified by univariate Cox analysis from the expression profile matrix using the coxph function in R. Subsequently, a total of 361 TCGA-OV samples were clustered by consensus clustering analysis based on the expression profiles of the 17 ferroptosis-related genes using “ConsensusClusterPlus” package [14]. 500 bootstraps containing 80% TCGA-OV patients were processed by k-means algorithm and euclidean distancing. The optimal subtypes were determined from 2 to 10 based on cumulative distribution function (CDF) and consensus matrix. Kaplan-Meier (KM) curve was plotted for each subtype in TCGA-OV cohort. Distributions of ferroptosis-related genes were compared using Kruskal-Wallis test and visualized using “ComplexHeatmap” package [15].

### Analysis of clinicopathological characteristics among the ferroptosis subtypes

The relationship of clinicopathological characteristics including age, Stage, Grade and ferroptosis subtypes was explored. Also, other immune subgroups including C1 (Wound Healing), C2 (IFN-γ Dominant), C3 (Inflammatory), and C4 (Lymphocyte Depleted) as well as “differenced”, “immunoreactive”, “mesenchymal”, and “proliferative” were collected from a previous study [16]. Distributions of clinicopathological features among the three subtypes were analyzed using ANOVA.

### Comparisons of mutation characteristics among the ferroptosis subtypes

Furthermore, the simple nucleotide variations (SNVs) data of TCGA-OV samples processed by MuTect2 software [17] were downloaded. Homologous recombination defects (HRD), mutant-allele tumor heterogeneity (MATH), and tumor mutation burden (TMB) were calculated using tmb function embedded in “maftools” package (version 2.8.05) [18] in R. Comparisons among the subtypes were performed using Kruskal-Wallis test.

### Analysis of immune characteristics among the ferroptosis subtypes

In TCGA-OV cohort, StromalScore, ImmuneScore, ESTIMATEScore, and TumorPurity were calculated using ESTIMATE algorithm to evaluate immune cell infiltration [19]. The abundance of 22 immune cells was measured with CIBERSORT (https://cibersort.‌stanford.edu/) [20]. Single-sample gene set enrichment analysis (ssGSEA) was employed to score 29 tumor microenvironment (TME)-related gene signatures as previously reported [21]. Based on a past study [22], we obtained 15 pathways related to immune pathways, stromal pathways, DNA damage repair, and oncogenic pathways, and the enrichment score was calculated by ssGSEA. Comparisons were performed by kruskal.test. Statistically significant was defined when *P* < 0.05.

### Identifying differential expressed genes (DEGs)

Under the criteria of false discovery rate (FDR) < 0.05 and |log2 (Fold Chage)| > 1, “limma” package [23] in R was used to filter the DEGs among the three ferroptosis subtypes. Differentially up-regulated genes in subtype C1 and down-regulated genes in subtype C3 were analyzed using Gene ontology (GO) and Kyoto Encyclopedia of Genes and Genomes (KEGG) enrichment analysis in “clusterProfiler” package [24].

### Development and verification of a risk model

Candidate DEGs with a potential impact on OV prognosis were first selected from a total of 3,384 DEGs under *P* < 0.01 using univariate Cox regression analysis, and their number was further reduced by LASSO Cox regression using “glmnet” package [25] in R. A risk model was developed applying stepwise multivariate regression analysis and stepAIC.

The formula for the risk score of the prognostic signature was:


$$\text{Risk}\;\text{score}=\sum_{i=1}^{i}\;\text{β}\;i\times {\text{EXP}}_{i}$$

where “β*i*” represents the Cox regression coefficient value of *i* gene and “EXP*i*” indicates the *i* gene expression level. The risk score was calculated for the samples in TCGA-OV cohort, and “timeROC” package [26] was used to conduct receiver operating characteristic (ROC) analysis with AUCs for 1, 3, and 5 year(s). Finally, the risk score was standardized to z-score to divide patients in TCGA-OV into low-risk group (zscore < 0) and high-risk group (zscore > 0). The “survminer” package [27] was used to determine the optimal cutoff, and KM curves of the two groups were plotted. Significant differences were defined by log-rank test. To validate the robustness of the risk model, patients in the validation cohorts were also divided into the two risk score subgroups according to the same method used to process the TCGA cohort. Then, KM curves and ROC curves were generated for the three subtypes in GSE102073 cohort and ICGC-AU cohort.

### Clinical performance of the risk score and prognosis analysis

For high- and low-risk groups, risk score distribution among the three subtypes was analyzed using the Kruskal.test and the Sankey diagram. KM curves were generated to compare the low- and high-risk groups in terms of several clinicopathological parameters (Stage II-IV and Grade G2-G3).

### Relationship of pathway characteristics and risk score

GSEA analysis was conducted on all candidate gene sets in KEGG and Hallmark database [28]. Meanwhile, the relationship between biological function of different samples and risk score was analyzed by calculating the scores of different functions in each OV sample using ssGSEA analysis in “GSVA” package [29]. The correlation heatmap of pathway and risk score was visualized by “ggcorrplot” package [30]. Distribution of ssGSEA scores between the two risk groups was analyzed using kruskal.test and visualized using “ComplexHeatmap” package.

### Assessment of immune cell infiltration between the high- and low-risk groups

Furthermore, we evaluated the immune cell infiltration between the two risk groups by ESTIMATE algorithm, and the differences were analyzed using wilcon.tests. The abundances of 22 immune cells were measured by CIBERSORT. The ssGSEA method was employed to score the 15 pathways-related gene signatures and 29 TME-related gene signatures. Comparisons were performed using kruskal.test. Statistical significance was defined when *P* < 0.05. Next, the correlation between immune cell infiltration, 22 immune cells and risk score was analyzed by “ggcorrplot” package.

### Prediction of patients’ response to chemotherapy and immunotherapy

Patients’ response to ICI therapy was evaluated using TIDE algorithm [31], with a high TIDE score indicating low response to ICI therapy. Immune checkpoints were obtained from a previous study [32]. The “ComplexHeatmap” package was used to compare immune checkpoint expression patterns between high- and low-risk groups. The relationship between risk score and immune checkpoints was analyzed using “ggcorrplot” package. Meanwhile, IC_50_ was calculated by the “pRRophetic” program [33] and compared in the two risk groups using wilcox.tests. Statistical significance was defined when *P* < 0.05.

### Cell culture and transfection

Two human ovarian cancer cell lines (SKOV3 and A2780) were purchased from the Cell Repository of the Chinese Academy of Sciences. McCoy’s 5A medium (HyClone, USA) containing 1% Penicillin-Streptomycin Solution (Procell Life Science and Technology Co., Ltd., China) and 10% fetal bovine serum (FBS, HyClone, USA) were used to culture SKOV3 cells. A2780 cells were cultured in Dulbecco’s Modified Eagle Medium (DMEM, HyClone, USA) supplemented with 1% Penicillin-Streptomycin Solution (Procell Life Science and Technology Co., Ltd., China) and 10% FBS (HyClone, USA). All the cells were placed in an incubator with 5% CO_2_ at 37°C.

The expression of PDP1 was inhibited using siRNA (GIMA Corporation, China). Initially, both cell lines (1 × 10^5^ cells/well) were seeded into 6-well plates containing 2-ml corresponding culture medium. Once the cells adhered to the surface, a mixture of 5 μl siRNA and 5 μl Lipo3000 (Invitrogen, USA) was added into each well. Subsequently, the cells were placed back into the incubator and the complete culture medium was replaced after incubation for 6 hours. Total RNA extraction and total protein extraction from the cells were performed 48 and 72 hours after transfection, respectively. Whether the knockdown of PDP1 was successful was analyzed by performing RT-qPCR and Western blot analysis. Cell phenotypic experiments were conducted 48 hours after the transfection to observe the changes in cellular phenotype after PDP1 knockdown.

The siRNA sequences used were as follows: si-negative control (NC): Sense: 5′-UCCUCCGAACGUGUCACGUTT-3′, Antisense: 5′-ACGUGACACGUUCGGAGAATT-3′; si-PDP1: Sense: 5′-TCAGTTCAATTCTCATGTT-3′, Antisense: 5′-TGAGACTTTGCTAGAGATT-3′. The primer sequences used were as follows: GAPDH: forward: 5′-GCAAATTCCATGGCACCGT-3′, reverse: 5′-TCGCCCCACTTGATTTTGG-3′, PDP1: forward: 5′-GTCCTTCCCATTCTGCAACC-3′, reverse: 5′-GAAACAGAGGAGGACCAAACA-3′.

### EdU assay

Forty-eight hours after the transfection, the two cell lines (6 × 10^3^ cells) were seeded into a 96-well plate per well. After cell adhesion, 100 μM of 2X EdU was added to each well and incubated for 2 hours. Next, phosphate-buffered saline (PBS) was used to wash the cells twice, followed by using 100 μL of general tissue fixative to fix the cells for 20 minutes. Next, 0.3% Triton X was added to lyse the cells for at least 10 minutes. Once the lysis was complete and cleared, the cells were added with a fluorescence staining reagent and then incubated for 40 minutes. Finally, after washing the cells at least twice with PBS, the nuclei were stained by adding Hoechst 33342. The experiment was conducted in triplicate.

### Wound-healing assay

After transfection for 48 hours, the two cell lines were observed to see if they covered 80% of a well of the 6-well plate. If the coverage was below 80%, cell culture was continued until the desired 80% coverage was achieved. To detect cell migration ability, 200 μL pipette tip was used to gently and uniformly scratch the surface of the central area in each well. Subsequently, the wells were washed by PBS twice and then the corresponding serum-free culture medium was renewed, and the plate was placed back into the cell culture incubator under the original conditions. After incubation for 24 hours, the 6-well plate was removed and images were captured using a microscope. Cell migration was determined by measuring the distance of wound closure on both sides of the scratch. The experiment was conducted in triplicate.

### Colony formation assay

After transfection for 48 hours, the cells (1,000 cells/well) were seeded into 6-well plates and cultured in complete medium for two weeks. After culturing, culture medium was discarded and the cells were washed with PBS three times. Subsequently, the cells were fixed with formaldehyde for 20 minutes and washed by PBS twice. 0.1% crystal violet was used to dye the cells for 20 minutes. The results were assessed by colony imaging and counting. This experiment was repeated three times.

### Transwell assay

After transfection for 48 hours, the cells were cultured for 24 hours in serum-free medium, then digested with trypsin, centrifuged, and washed with PBS twice. The cells were resuspended in 200 μL of serum-free medium and counted to ensure that there were approximately 8 × 10^3^ cells per upper chamber. For the invasion assay, the bottom surface of the chamber was pre-coated with matrix gel and the lower chamber was added with 500 μL of complete medium containing 10% FBS. After incubation for 24 hours, the chamber was washed twice with PBS, and non-invaded cells were removed using cotton swab. Next, 4% paraformaldehyde was used to fix the chamber for 20 minutes. The cells were dyed by 0.1% crystal violet for 20 minutes and washed with PBS. The chamber was placed under a 200X magnification microscope for imaging and cell counting. This experiment was repeated three times.

### Data availability statement

The data that support the findings of this study are openly available in The Cancer Genome Atlas (TCGA, https://portal.gdc.cancer.gov/), Gene-Expression Omnibus (GEO, https://www.ncbi.nlm.nih.gov/geo/), and International Cancer Genome Consortium (ICGC, https://icgc.org). The experimental data can be obtained from the corresponding author upon reasonable request.

## RESULTS

### Classification of three ferroptosis subtypes

**Figure 1:**
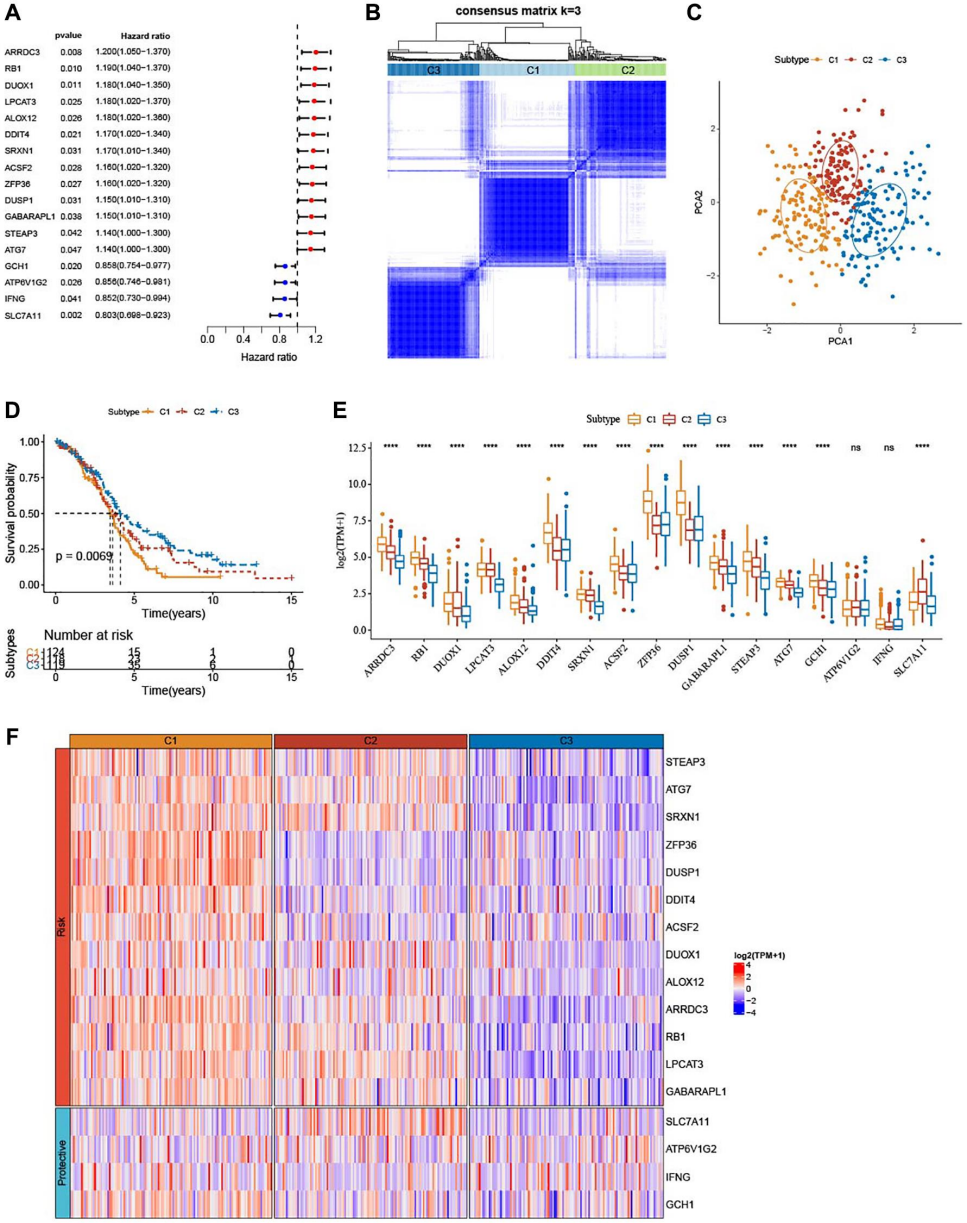
**Identification of three ferroptosis-based subtypes.** (**A**) Forest plots for a total of 17 ferroptosis-related genes in TCGA-OV cohort analyzed by univariate Cox regression study. (**B**) Three clusters (k = 3) were classified by consensus matrix heatmap defining. (**C**) The three subtypes showed distinct separation, shown by PCA analysis. (**D**) The three subtypes in TCGA-OV cohort were plotted for Kaplan-Meier curves. (**E**) Alterations of 17 genes related to ferroptosis in TCGA-OV cohort. (**F**) Heatmap portrayed the distributions of the genes related to ferroptosis among three subtypes. ^ns^*P* > 0.05; ^****^P < 0.0001.

A total of 17 prognostic genes were selected by univariate Cox regression analysis from TCGA-OV cohort (Figure 1A). A total of 361 OV patients were clustered by these genes using consensus clustering analysis. According to the CDF Delta area, when cluster = 3, clustering effect was relatively stable (Supplementary Figure 1). As shown in Figure 1B, consensus matrix k = 3 was determined to classify three subtypes of OV. Principal components analysis (PCA) presented distinct separation among the three subtypes (Figure 1C). From KM curves, it could be observed that subtype C3 had the optimal prognosis and subtype C1 had the worse survival in TCGA-OV cohort (*P* = 0.0069) (Figure 1D). Furthermore, the expression of the 17 genes was measured (Figure 1E). Except ATP6V1G2 and IFNG, subtype C1 had most of significantly upregulated ferroptosis-related genes. Similarly, the heatmap displayed the distributions of ferroptosis-related genes among the three subtypes. We found that these ferroptosis-related genes were remarkably upregulated in subtype C1 but downregulated in subtype C3 (Figure 1F).

### Association of clinicopathologic characteristics among the three ferroptosis subtypes

**Figure 2:**
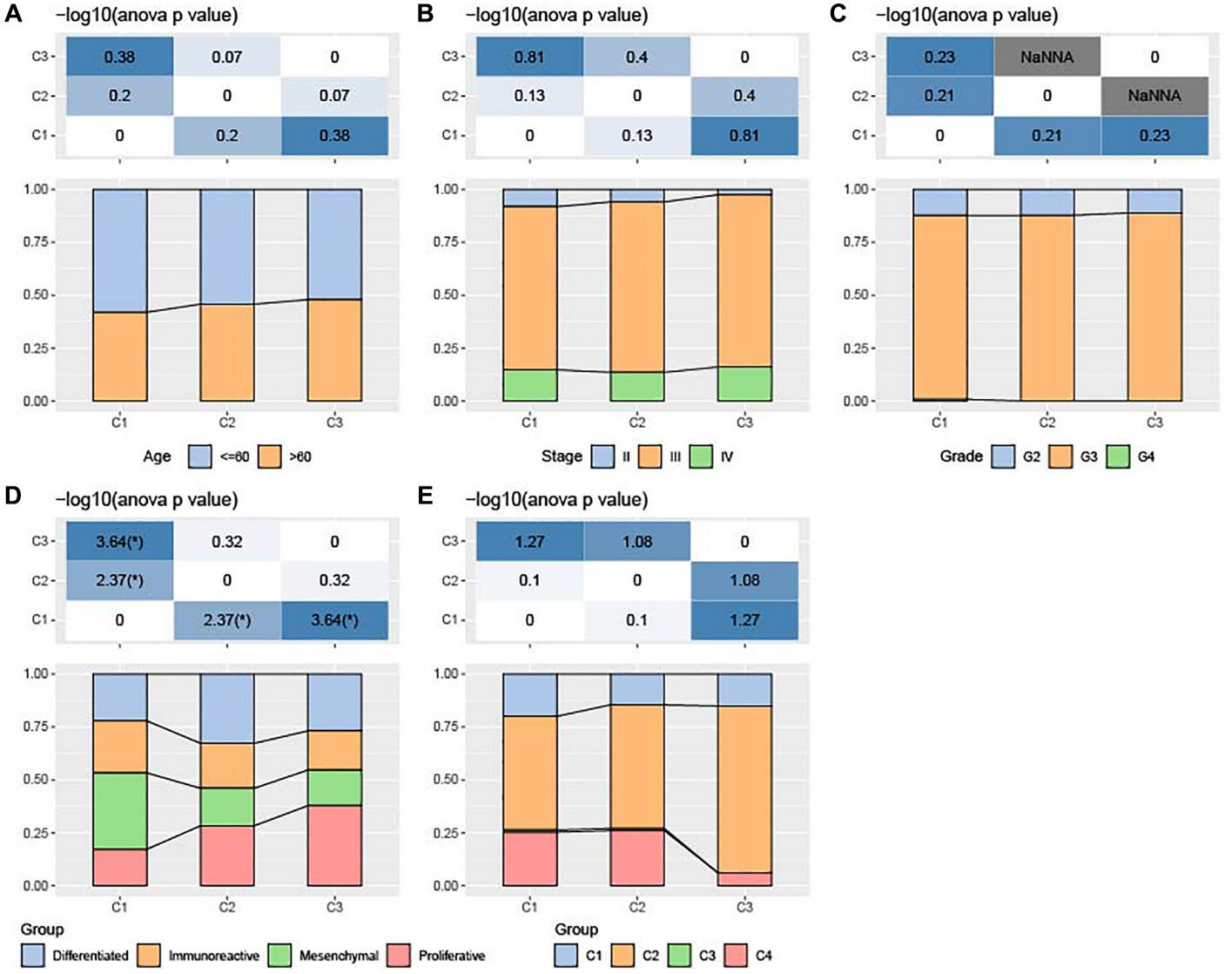
**Association of clinicopathologic characteristics among ferroptosis-based subtypes in TCGA-OV cohort.** (**A**–**C**) Distributions of age, Stage and Grade among the three subtypes. (**D**) Distributions of differenced, immunoreactive, mesenchymal, and proliferative subtypes among the three subtypes. (**E**) Alterations of immune subtypes of C1, C2, C3, and C4 in the three subtypes.

The three ferroptosis subtypes in TCGA-OV cohort showed no significant difference in the clinicopathological features such as Grade, age or Stage (Figure 2A–2C). Further, we collected other subtypes (differenced, immunoreactive, mesenchymal, and proliferative) and immune subtypes (C1, C2, C3, and C4) from a previous study. Subtype C1 and subtype C2 had higher proportion of immune subtype C4 (Lymphocyte Depleted); subtype C3 exhibited higher proportion of immune subtype C2 (IFN-γ Dominant); the quantity of mesenchymal cells was significantly more in subtype C1 than in subtype C3 (Figure 2D, 2E).

### Mutation characteristics among the ferroptosis subtypes

**Figure 3:**
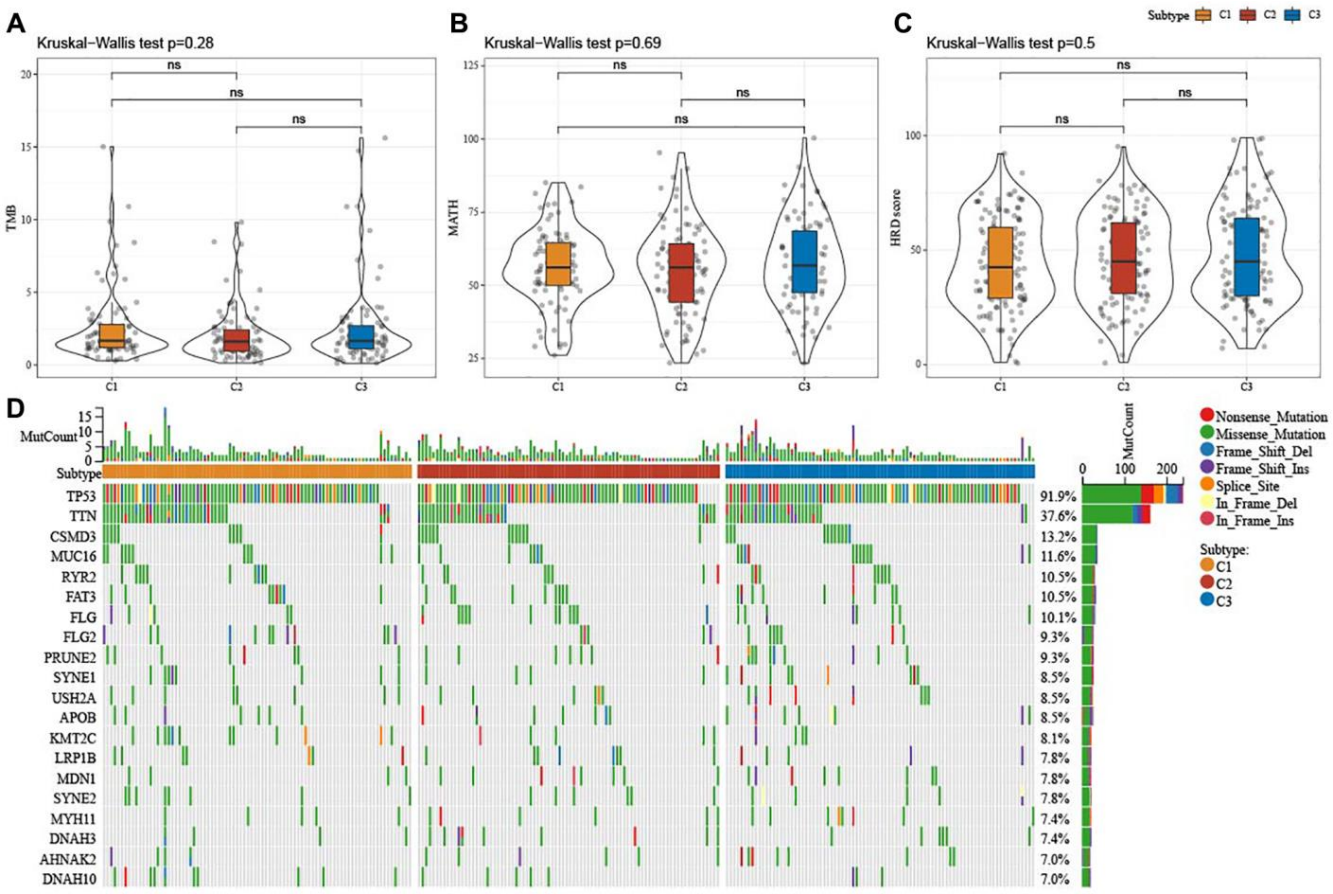
**Mutation characteristics among ferroptosis-based subtypes in TCGA-OV cohort.** (**A**–**C**) Alterations of TMB, mutant-allele tumor heterogeneity, and homologous recombination defects in TCGA-OV cohort. (**D**) Mutation frequencies of top 20 somatic mutations in TCGA-OV cohort. ns represents *P* > 0.05.

Figure 3A–3C displayed no significant differences in TMB, MATH, and HRD among the three subtypes (*P* > 0.05). Meanwhile, analysis on the mutation characteristics of top 20 mutated genes in each subtype showed higher mutation frequencies of TP53 (91.9%) and TTIN (37.6%) (Figure 3D).

### Immune characteristics among the ferroptosis subtypes

**Figure 4:**
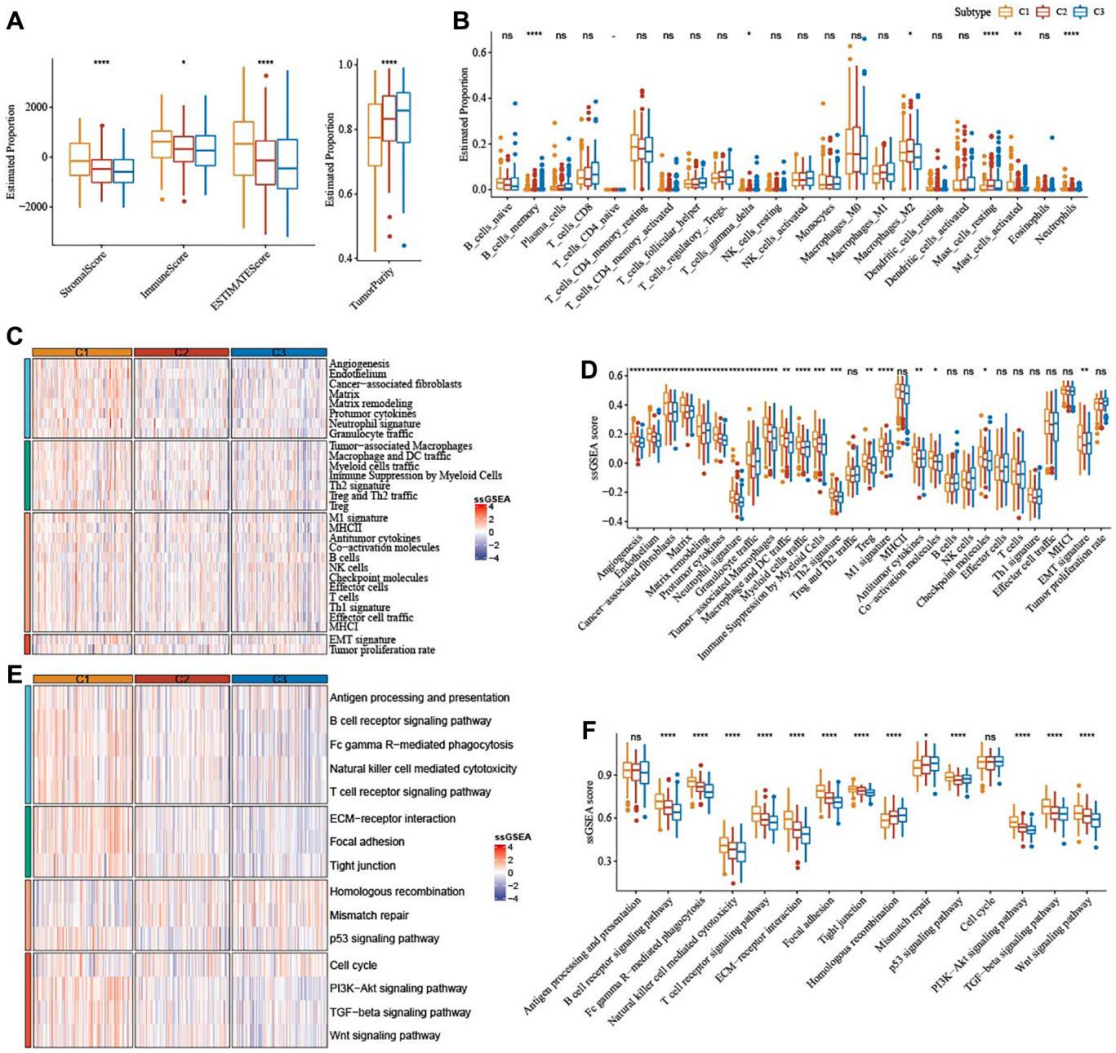
**Immune characteristics among the subtypes correlated with ferroptosis in TCGA-OV cohort.** (**A**) The immune infiltration score of the three subtypes were shown in box plot. (**B**) Abundance of immune cells infiltration of 22 immune cells among three subtypes. (**C**) 29 TME-related gene signatures among three subtypes were shown in heatmap. (**D**) 29 gene signatures related to TME were shown in box plots. (**E**) 15 pathways-correlated gene signatures among three subtypes were shown in the heatmap. (**F**) Box plots of 15 pathways-related gene signatures among three subtypes. ns represents *P* > 0.05; ^*^P < 0.05, ^**^P < 0.01, ^***^P < 0.001, and ^****^P < 0.0001.

The results from ESTIMATE showed that StromalScore (*P <* 0.0001), ImmuneScore (*P <* 0.05), and ESTIMATEScore (*P <* 0.0001) were remarkably higher in subtype C1, but TumorPurity (*P <* 0.0001) was lower in subtype C1 when compared to subtype C2 and subtype C3 (Figure 4A). Significant alterations in the abundance of immune cells infiltration among the three subtypes were analyzed, and we found that subtype C1 had abundant Macrophages_M2 (Figure 4B). SsGSEA scores for 29 TME-related gene signatures demonstrated that matrix, matrix remodeling, angiogenesis, cancer-associated fibroblasts (CAF), endothelium, protumor cytokines, and EMT signature were enriched in subtype C1 (Figure 4C, 4D). Moreover, subtype C1 had distinctly higher scores of Wnt signaling pathway, PI3K-Akt signaling pathway, TGF-β signaling pathway (Figure 4E, 4F).

### Identification of DEGs

**Figure 5:**
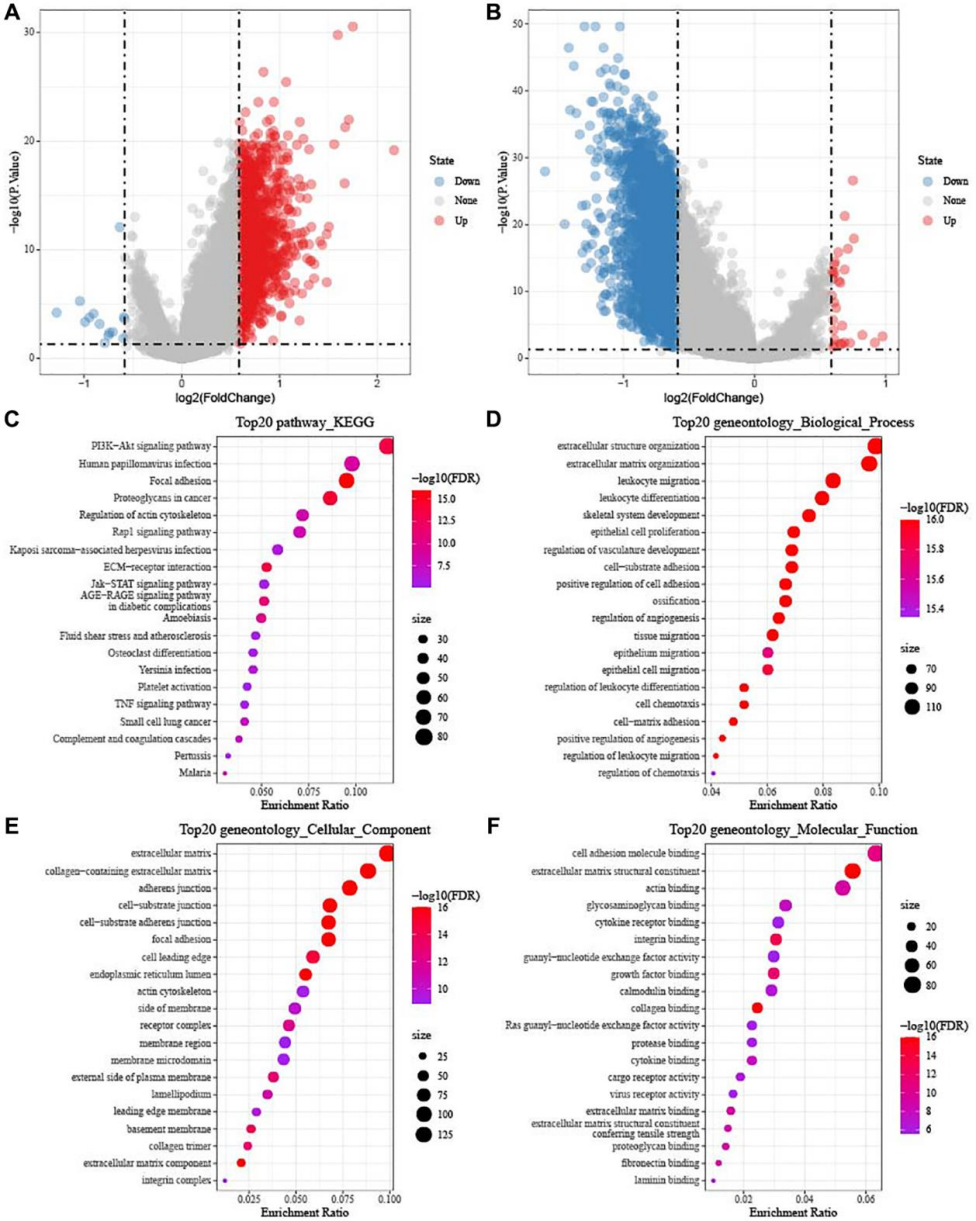
**Identification of DEGs in TCGA-OV cohort.** (**A**) Volcano diagram shows DEGs between subtype C1 and others. (**B**) The DEGs between subtype C3 and others were shown in volcano diagram. Blue dots mean genes with downregulated expression and red dots mean genes with upregulated expression. (**C**–**F**) Functional KEGG and GO enrichment analysis between subtype C1 and others.

Furthermore, we identified 1420 DEGs from subtype C1 (including 1406 upregulated and 14 downregulated genes) (Figure 5A), 51 DEGs from subtype C2 (including 19 upregulated and 32 downregulated genes), and 3120 DEGs from subtype C3 (including 30 upregulated and 3090 downregulated genes) (Figure 5B). Next, differentially up-regulated genes in subtype C1 and down-regulated genes in subtype C3 were subjected to functional GO and KEGG pathway enrichment analysis. As shown in Figure 5C, focal adhesion and proteoglycans, PI3K-Akt signaling pathway in cancer were significantly enriched in subtype C1. Figure 5D–5F showed the top 20 enriched GO terms (BP, CC, and MF). As for BO, subtype C had enriched regulation of extracellular matrix organization, angiogenesis, and positive regulation of cell adhesion.

### Development and verification of a risk model

**Figure 6:**
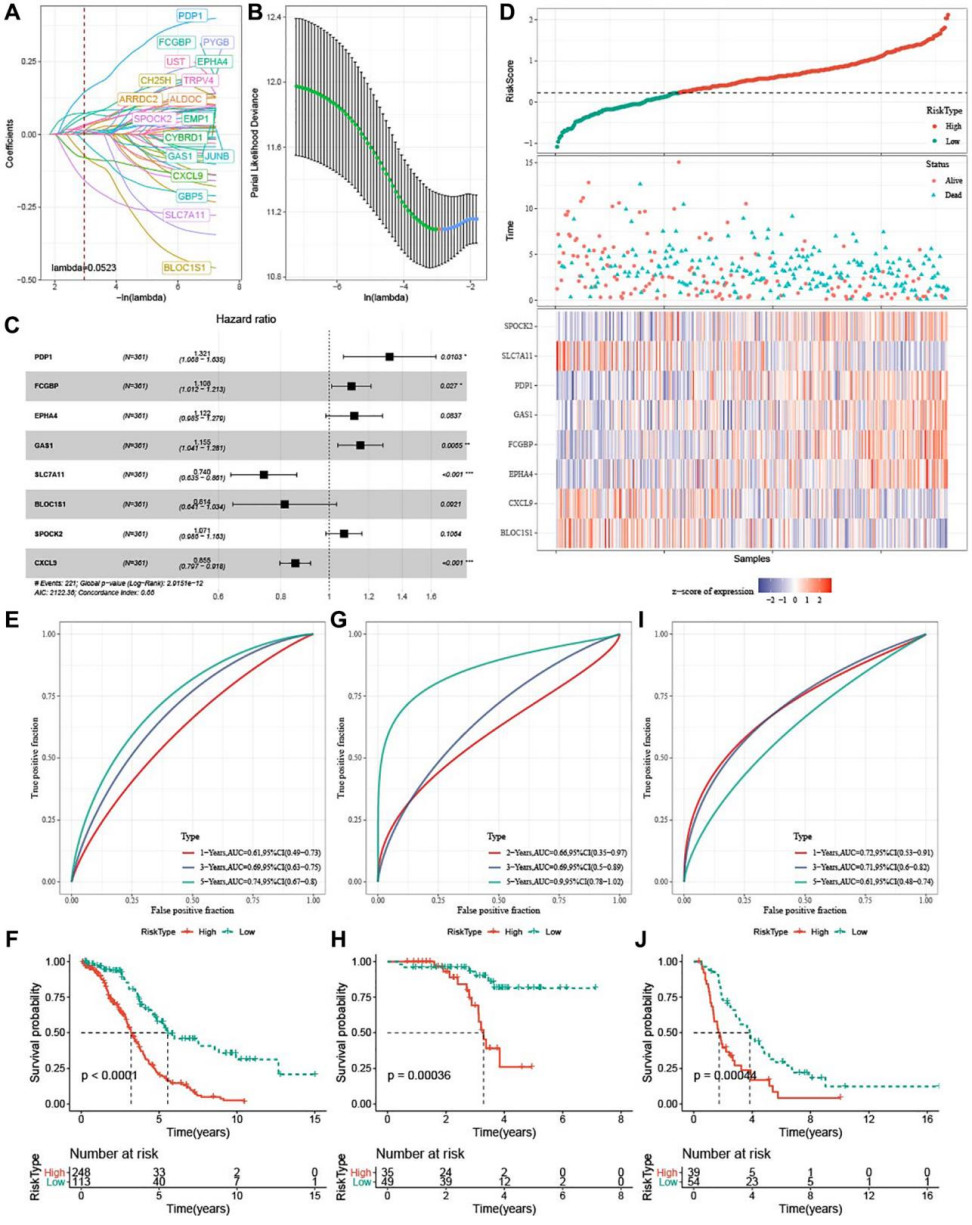
**Development and verification of ferroptosis-based risk model.** (**A**) Mutual increase between independent variable coefficients and lambda. A total of 18 genes were detected when lambda = 0.052. (**B**) The confidence interval under each lambda determined by 10-fold cross validation. (**C**) Forest plots of 8 ferroptosis-related prognostic genes TCGA-OV cohort. (**D**) Distributions of ferroptosis-related prognostic genes, risk score, survival status in TCGA-OV cohort. (**E**, **F**) ROC curves with AUCs and Kaplan-Meier curves plotted from patients with high and low risk in TCGA-OV cohort. (**G**, **H**) ROC curves with AUCs and Kaplan-Meier curves plotted from patients with low and high risk in GSE102073 cohort. (**I**–**J**) ROC curves with AUCs and Kaplan-Meier curves plotted from patients with low and high risk in ICGC-AU cohort.

A total of 52 (47 risk genes and 5 protective genes) genes with greater impact on prognosis were identified from a total of 3,384 DEGs. LASSO Cox regression was carried out to compress gene number in the risk model. Figure 6A displayed mutual increases in both the number of independent variable coefficients close to zero and the lambda. See Figure 6B for confidence interval under each lambda in 10-fold cross-validation. Using stepwise multivariate regression analysis and stepAIC under lambda = 0.052, 8 ferroptosis-related genes (PDP1, FCGBP, EPHA4, GAS1, SLC7A11, BLOC1S1, SPOCK2, and CXCL9) affecting OV prognosis were selected (Figure 6C). The risk score was calculated with the formula: risk score = + 0.279 × PDP1 + 0.102 × FCGBP + 0.115 × EPHA4 + 0.144 × GAS1 −0.302 × SLC7A11-0.205 × BLOC1S1 + 0.068 × SPOCK2 −0.156 × CXCL9. We found that high-risk patients in TCGA dataset showed a poor prognostic outcome (Figure 6D). In TCGA cohort, the survival of high-risk patients was worse than low-risk patients (*P* < 0.0001), with 1-year AUC of 0.61, 3-year AUC of 0.69, and 5-year AUC of 0.74 (Figure 6E, 6F). The model robustness was verified using GSE102073 cohort and ICGC-AU cohort because 1-year, 3-year and 5-year in GSE102073 cohort was 0.66, 0.69, and 0.90, respectively, and in ICGC-AU cohort was 0.72, 0.71, and 0.61, respectively (Figure 6G–6J).

### Clinical associations and prognosis analysis of the risk score

**Figure 7:**
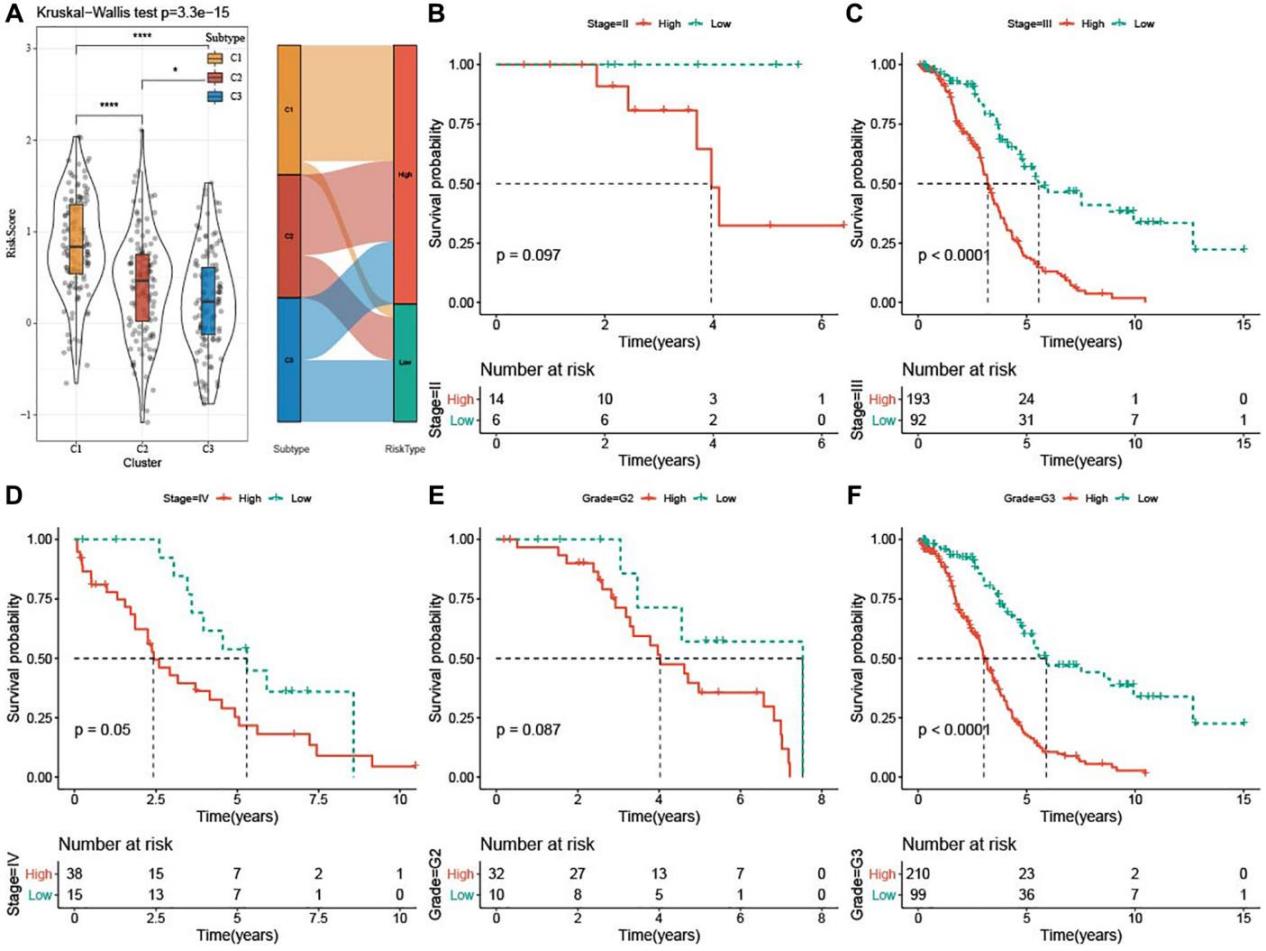
**Clinical associations and prognosis analysis of risk score in TCGA-OV cohort.** (**A**) Risk score among the three subtypes was shown in violin plots and Sankey diagram represented the distribution of three subtypes in low-risk and high-risk groups. (**B**–**F**) Kaplan-Meier curves for patients with different clinicopathological characteristics (Stage II–IV and Grade G2-G3) in the two risk groups. ^*^P < 0.05 and ^****^P <0.0001.

The risk score was the highest in subtype C1 and the lowest in subtype C3. Additionally, most high-risk patients belonged to subtypes C1 and C2 (Figure 7A). Furthermore, survival analysis showed that high-risk patients with Stage III-IV and Grade G3 exhibited dismal prognosis (Figure 7B–7F).

### High risk was associated with EMT pathway

**Figure 8:**
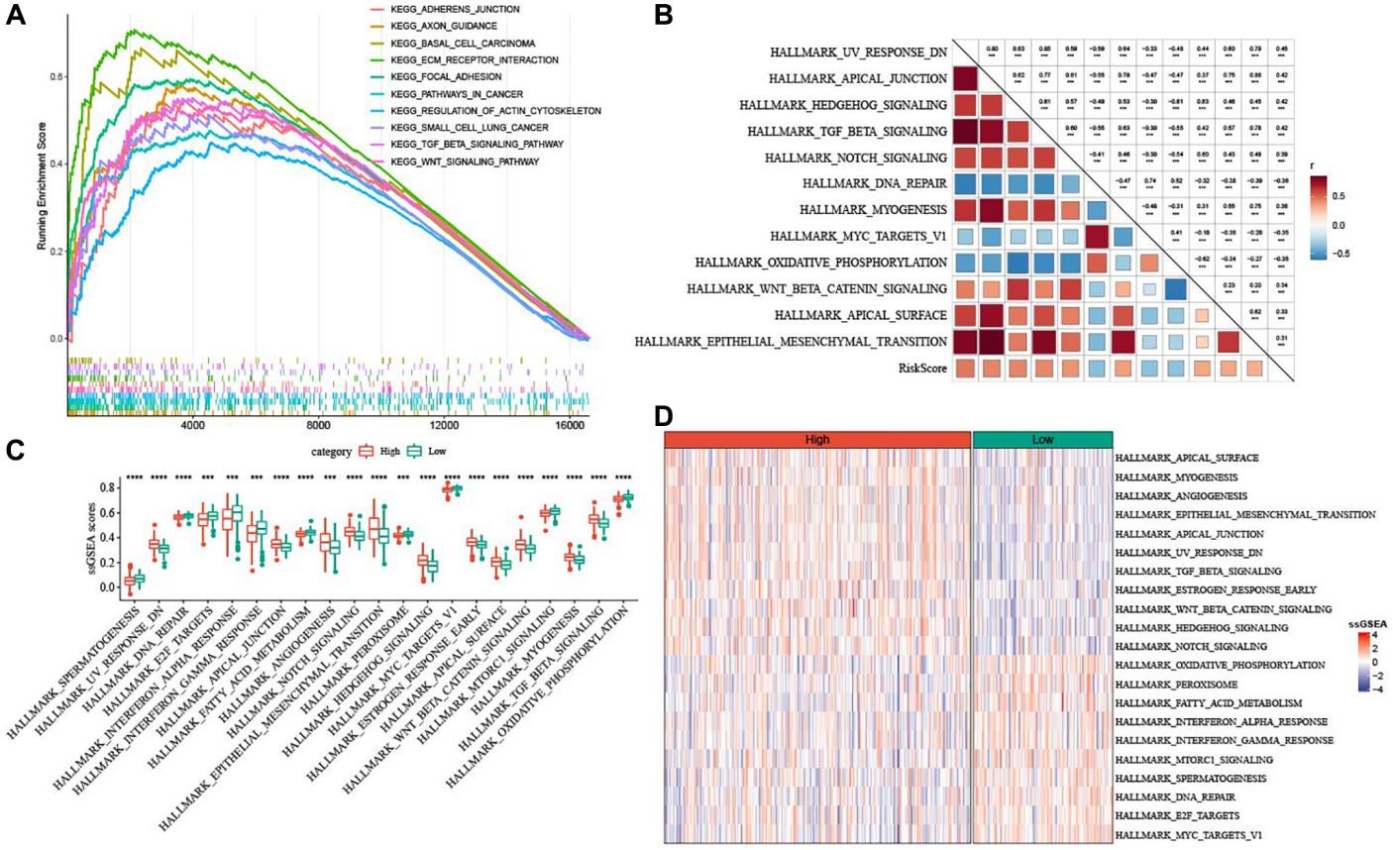
**Pathway characteristics of high- and low-risk groups.** (**A**) GSEA analysis between the two risk groups based on KEGG database. (**B**) Heatmap for the correlation between hallmark pathways and risk score. (**C**) Box plots of differential pathway ssGSEA scores between the two risk groups. (**D**) Heatmap of differential pathways between the two risk groups. ^***^P < 0.001, and ^****^P < 0.0001.

To clarify whether there were differentially activated pathways in high- and low-risk groups, we performed GSEA analysis using KEGG database and Hallmark gene sets. KEGG_BASAL_CELL_CARCINOMA, KEGG_ECM_RECEPTOR_INTERACTION, KEGG_TGF_BETA_SIGNALING_PATHWAY and KEGG_WNT_SIGNALING_PATHWAY (two EMT-related pathways), KEGG_FOCAL_ADHESION were enriched in the low-risk group (Figure 8A). In addition, high risk was positively associated with pathways involved in EMT process, such as HALLMARK_WNT_SIGNALING, and HALLMARK_TGF_BETA_SIGNALING, HALLMARK_NORCH_SIGNALING, HALLMARK_EPITHELIAL_MESENCHYMAL_TRANSITION (Figure 8B). As illustrated in Figure 8C, 8D, high-risk score group had significantly enriched EMT-related pathways.

### Potential immune characteristics of the two risk groups

**Figure 9:**
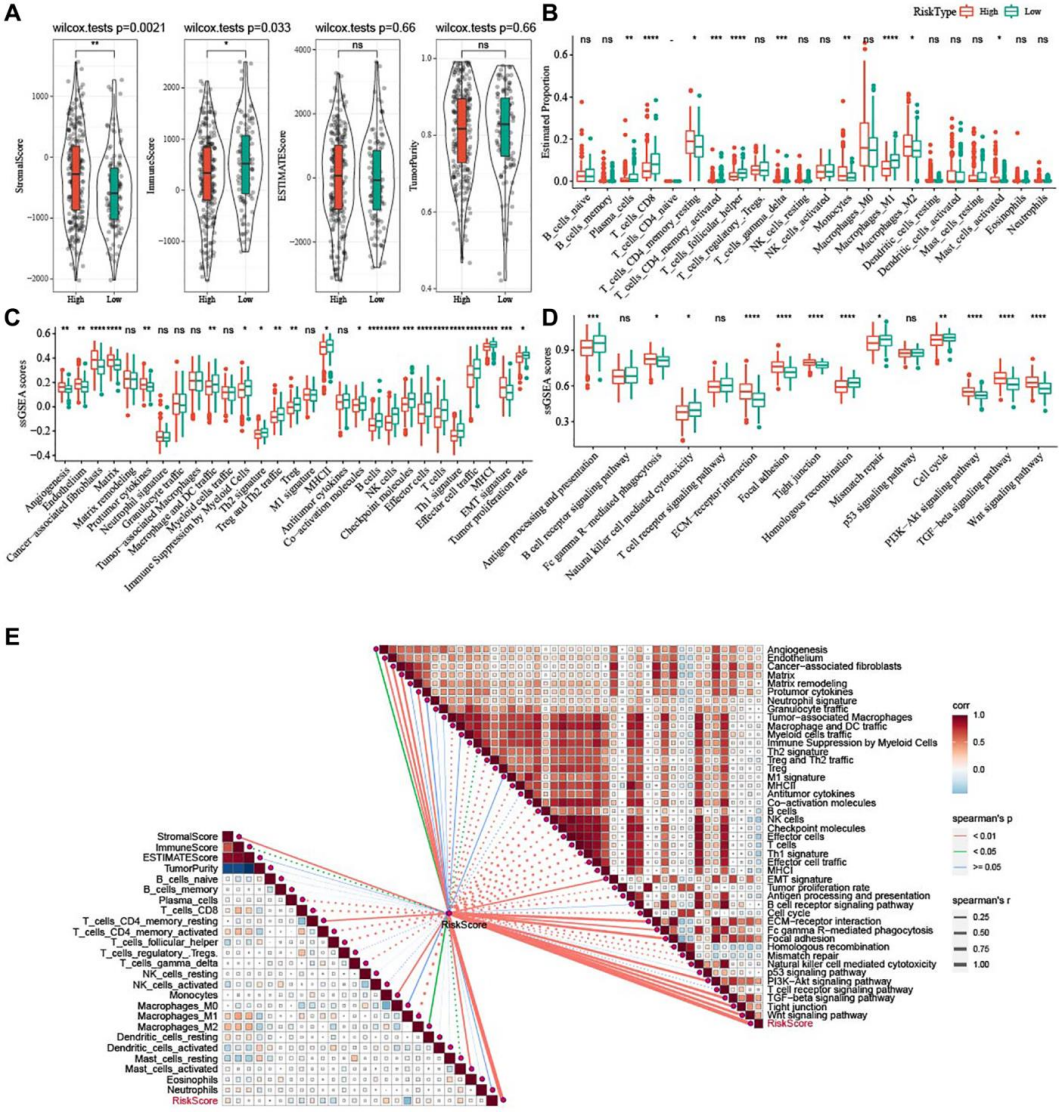
**Potential immune characteristics of high- and low-risk groups.** (**A**) Violin plots of ImmuneScore, StromalScore, ESTIMATEScore, and TumorPurity between the two risk groups in TCGA-OV cohort. (**B**) Box plots of 22 immune cells scores between the two risk groups in TCGA-OV cohort. (**C**) 29 TME-related gene signatures between the two risk groups in TCGA-OV cohort were shown in box plots. (**D**) 15 pathway-related gene signatures between high- and low-risk groups in TCGA-OV cohort were shown in box plots. (**E**) Correlation analysis between immune characteristics and risk score. ^ns^*P* > 0.05; ^*^P < 0.05, ^**^P < 0.01, ^***^P < 0.001, and ^****^P < 0.0001.

To elucidate the alterations in immune microenvironment, we determined the immune infiltration between the two risk groups. Figure 9A revealed that patients with low risk had higher ImmuneScore (*P* = 0.033) but lower StromalScore (*P* = 0.0021). The T_cells_follicular_Tregs, Macrophages_M1, T_cells_CD8, Plasma_cells, T_cells_CD4_memory_activated accumulated noticeably more in patients with a low risk, while high-risk patients had more Macrophages_M2 (Figure 9B). Furthermore, the score of 29 TME-related gene signatures in the two risk groups was calculated, and angiogenesis, CAF and EMT were highly enriched in patients with a high risk, whereas anti-tumor cytokines, NK cells, and T cells were more enriched in low-risk patients (Figure 9C). As shown in Figure 9D, EMT-related pathways, including PI3K-Akt signaling pathway, Wnt signaling pathway, and TGF-beta signaling pathway, were significantly activated in high-risk patients, while some pathways such as homologous recombination, antigen processing and presentation, natural killer cell-mediated cytotoxicity, mismatch repair were apparently activated in low-risk patients. Further correlation analysis demonstrated that the risk score was positively related to StromalScore, Macrophages_M2, Mast_cells_activated, angiogenesis, fibroblasts, EMT signature, and EMT-related pathways (Figure 9E).

### Prediction of patients’ responses to immunotherapy and chemotherapy

**Figure 10:**
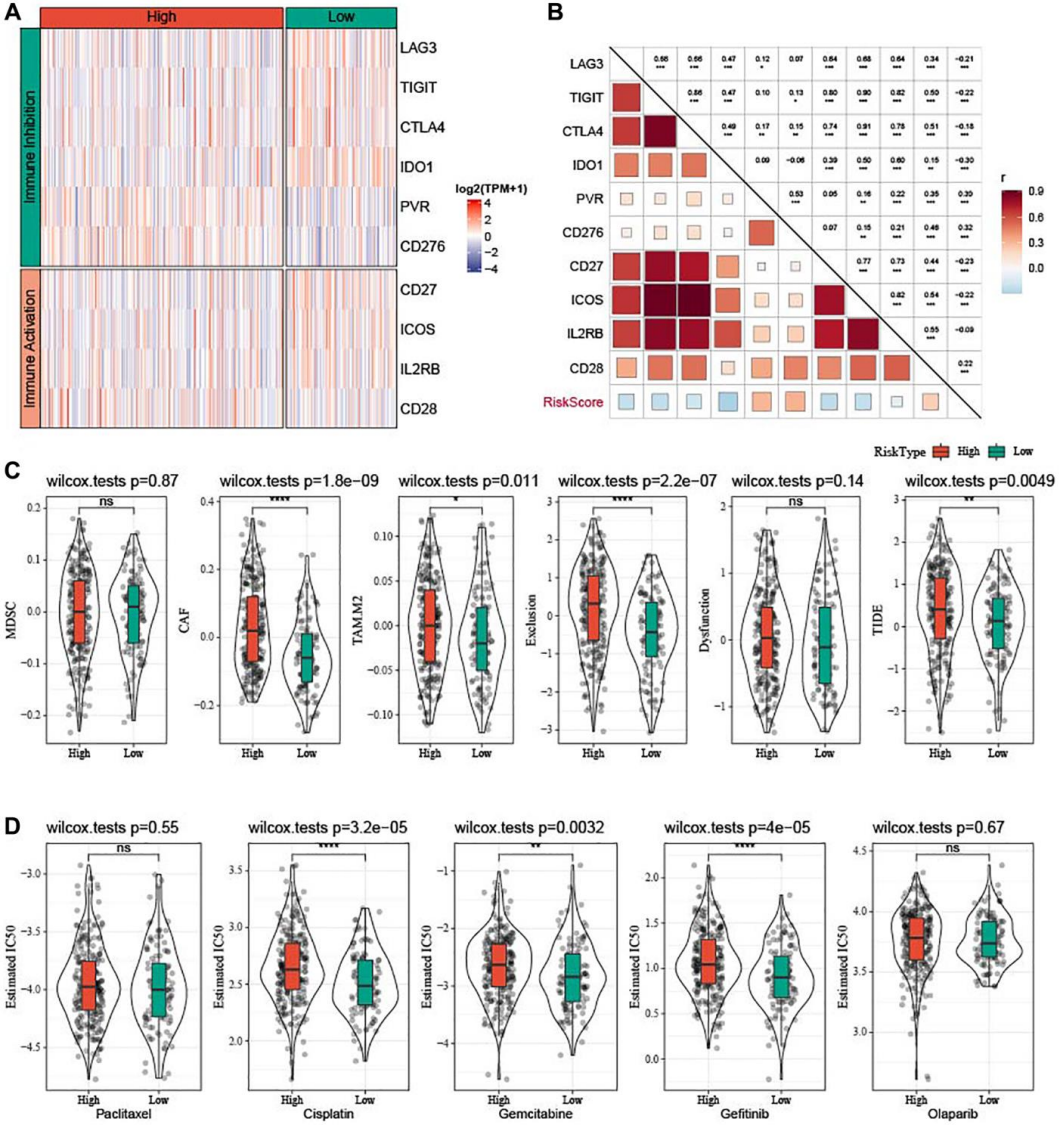
**Prediction of responsiveness to immunotherapy and chemotherapy in TCGA-OV cohort.** (**A**) Heatmap of differential expressed immune checkpoints between the two risk groups. (**B**) Correlation analysis between differential expressed immune checkpoints and risk score. (**C**) Violin plots of MDSC, CAF, TIDE, TAM.M2, exclusion, and dysfunction. (**D**) Estimated IC50 values for traditional chemotherapy drugs Paclitaxel, Cisplatin, Gemcitabine, Gefitinib, and Olaparib were shown in violin plots. ^ns^*P* > 0.05; ^*^P < 0.05, ^**^P < 0.01, ^***^P < 0.001, and ^****^P < 0.0001.

Differences in response of high-risk patients to anti-tumor therapies were analyzed. Differential analysis of immune checkpoints (Figure 10A) revealed that inhibitory immune checkpoints such as LAG3, TIGIT, CTLA4, and IDO1 were mainly upregulated in low-risk patients, while PVR and CD276 were upregulated in high-risk patients. CD27, ICOS, and IL2RB were high-expressed in low-risk samples, whereas CD28 was high-expressed in high-risk samples. The risk score showed positive correlation with PVR, CD276, and CD28 and negative correlation with LAG3, TIGIT, CTLA4, IDO1, CD27, ICOS, and IL2RB (Figure 10B). Patients with low risk exhibited lower CAF (*P* = 1.8e-09), TAM.M2 (*P* = 0.011), exclusion score (*P* = 2.2e-07), and TIDE score (*P* = 0.0049), indicating that they might have less escape from immune elimination (Figure 10C). Moreover, we also evaluated the response to traditional chemotherapy drugs (Paclitaxel, Cisplatin, Gemcitabine, Gefitinib, and Olaparib) of all the TCGA-OV patients, and found a higher sensitivity of low-risk patients to Cisplatin, Gemcitabine, and Gefitinib (Figure 10D).

### PDP1 promoted the proliferation, invasion, and migration of OV cells

**Figure 11:**
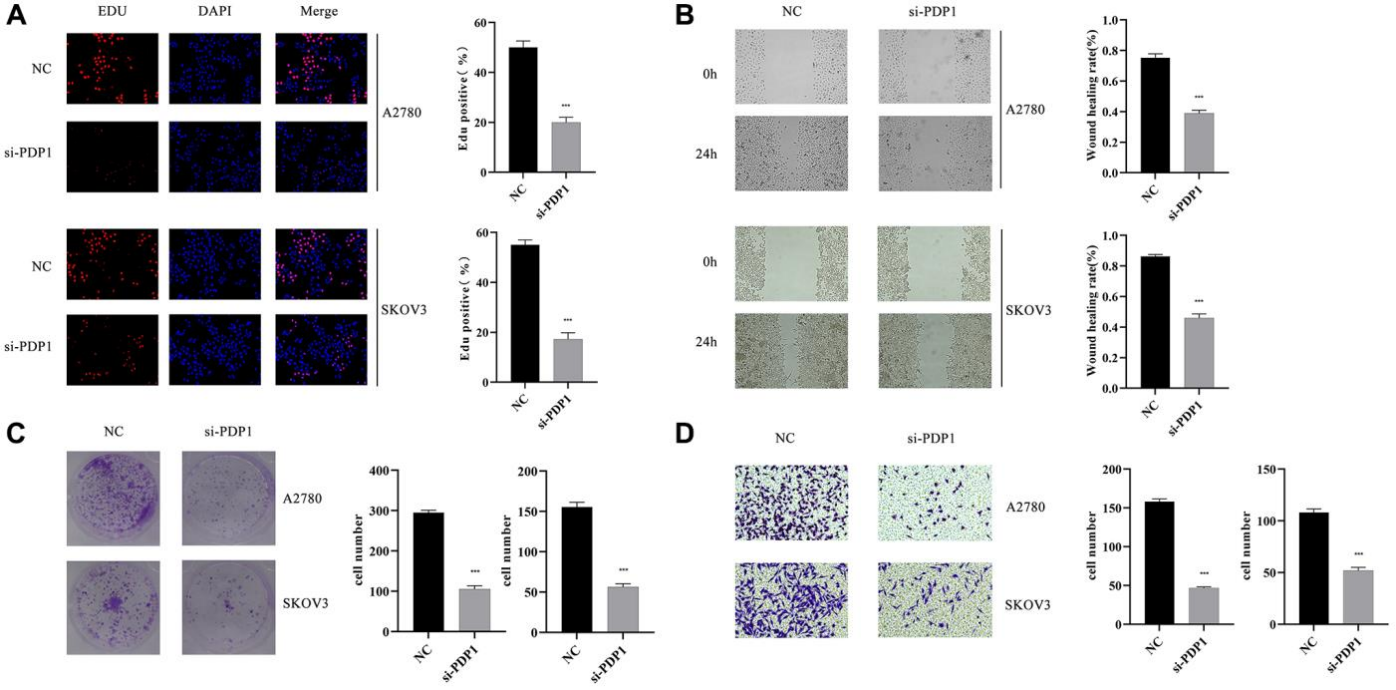
**The impact of PDP1 knockdown on proliferation, invasion, and migration was investigated in two cell lines, SKOV3 and A2780.** (**A**) EdU assay revealed a reduced number of proliferating cells in the si-PDP1 group compared to controls. (**B**) Wound-Healing assay demonstrated a decreased migration ability of cells in the si-PDP1 group. (**C**) Colony formation assay showed a reduced number of cell colonies formed in the si-PDP1 group. (**D**) Transwell assay indicated a decreased invasion ability of cells in the si-PDP1 group.

The PDP1 gene in the two OV cell lines was knocked down, and we conducted multiple phenotypic experiments to investigate its impact on OV cancer cells. The EdU assay showed that the si-PDP1 group had fewer proliferating cells when compared to the negative control group, indicating reduced proliferation of OV cells (*p* < 0.001, Figure 11A). In the colony formation assay, the si-PDP1 group formed significantly fewer cell colonies than the control group, which showed reduced level of cancer cell proliferation from a macroscopic perspective (*p* < 0.001, Figure 11C). The wound-healing assay revealed that compared to the negative control group, the ability of si-PDP1 group cells to migrate declined (*p* < 0.001, Figure 11B). After PDP1 knockdown, the transwell assay demonstrated a significant decrease in the number of cells invading the lower surface of the chamber (*p* < 0.001, Figure 11D). Collectively, our findings indicated that proliferation, invasion, and migration of OV cells could be promoted by PDP1.

## DISCUSSION

High-grade serous OV is the most common subtype of OV. In serous OV cells, disrupted iron metabolism leads to overload of iron, which provides a basis for the occurrence of ferroptosis. Thus, analysis on the role of ferroptosis in TME of serous OV may reveal a potential therapeutic approach to the clinical treatment of high-grade serous OV. Henein, we classified three subtypes of OV based on ferroptosis, and observed significant differences in tumor immune microenvironment and prognosis among the three subtypes. Next, using 8 ferroptosis-related genes, a prognostic model was developed to accurately evaluate the response to immunotherapy and chemotherapy and the prognosis.

Genomic alterations are predominant mediators that affect normal activity of genes and lead to the disrupting of homeostasis in malignant tumors. As a tumor suppressor, TP53 is mutant in 97% of high-grade serous OV cases [34], which also indicates that TP53 somatic mutation is a crucial factor for OV initiation [35]. It has been reported that TP53 mutations contribute to tumor progression through changing cellular iron acquisition and metabolism and are more sensitive to ferroptosis. Hence, inducing ferroptosis may represent a potential treatment for tumors with distinct TP53 mutation [36]. TTN is a frequently altered gene in gynecological tumors [37]. A recent study has demonstrated that both TP53 and TTN are the most somatic mutant genes in the ICGC cohort and TCGA cohort. Meanwhile, patients with TP53 and TTN mutations have obviously elevated TMB than those of wild-type patients [38]. Additionally, Wang and colleagues have also revealed that the most frequently altered genes are TP53 (88%) and TTN (34%), which are also related to the risk groups based on ferroptosis in OV patients [39]. The current study analyzed the somatic mutations in each subtype, and the results showed that TP53 (91.9%) and TTN (37.6%) exhibited the highest mutation frequencies. Our analysis of somatic mutation frequency indicated that higher mutation frequencies in TP53 and TTN might be implicated in the development and progression of high-grade serous OV through suppressing ferroptosis-mediated process.

TME contains various molecular and cellular factors and plays crucial roles in biological processes of tumor. Immune cell infiltration is an important indicator of the TME in tumor. This study evaluated the immune landscape among the three ferroptosis subtypes, and found that subtype C1 had abundant Macrophages_M2. Macrophages are innate immune cells that can digest foreign substances and remove harmful substances, such as cellular fragments and tumor cells. According to their response to microenvironmental stimuli, macrophages are divided into alternatively activated (M2) and classically activated (M1) macrophages [40]. Macrophages_M2, alternatively known as TAMs, loses its phagocytosis of tumor cells, promote tumor cell escape, leading to the spread of tumor cells to other tissues and organs [41]. Previous study has confirmed that Macrophages_M2 infiltration into tumor islets could cause poor prognostic outcomes in non-small-cell lung cancer [42] and poor response rate of ICI therapies in patients with prostate cancer [43]. Hence, we suspected that the abundance of Macrophages_M2 infiltration was closely related to a poor OV prognosis in subtype C1. Additionally, in tumor stroma, CAFs is one of the most important cellular components that could be recruited and reprogrammed by cancer cells to promote tumor cell growth and spread. CAFs are involved in TME of OV and promote the progression of OV [44]. Moreover, the coordination of the actin and septin networks in CAFs is essential for matrix remodeling, angiogenesis, and tumor growth [45]. Collectively, increased matrix, matrix remodeling, and angiogenesis caused by Macrophages_M2 infiltration and CAFs might be related to the poor prognosis in subtype C1.

Tumor tissues comprise of mesenchymal cells, immune cells, stromal cells as well as tumor cells. These noncancerous cells are considered as contaminants that affect tumor purity. Gong et al. demonstrated that lower tumor purity in gastric cancer patients is closely related to poor prognosis, upregulated EMT and stemness pathways, more immune cell infiltration such as Tregs, M1 and M2 macrophages and elevated levels of immune checkpoints and chemokines as well as immunosuppression [46]. Mao et al. also revealed that lower tumor purity in colon cancer is associated with unfavorable prognosis, increased TMB, intense immune phenotype, and high expression of immune checkpoints [47]. This study found that subtype C1 had an unfavorable prognosis and exhibited higher StromalScore, ImmuneScore, and ESTIMATEScore, but lower tumor purity. Moreover, mesenchymal cells were significantly higher in subtype C1 than C3. We therefore speculated that more mesenchymal cells and increased Macrophages_M2 infiltration affected tumor purity, which served as crucial factors in tumor progression and caused a dismal prognosis to patients with subtype C1.

During EMT process, epithelial cells acquire mesenchymal features and function critically in embryogenesis, tissue regeneration and tumor progression [48]. Activating EMT induces cell polarity loss, cell-cell junction breakage, basement membrane degradation and extracellular matrix (ECM) reorganization. EMT can be activated by signaling pathways, including Wnt signaling pathway, NOTCH pathway, PI3K-Akt signaling pathway, TGF-beta signaling pathway [49]. Interestingly, except intracellular signaling pathways, stromal components in the tumor microenvironment such as CAFs and TAMs that can secrete TGF-beta, contributing to the activation of EMT [49]. Consistent with these findings, we found enriched EMT signature and EMT-related pathways including TGF-β signaling pathway, Wnt signaling pathway, and PI3K-Akt signaling pathway in subtype C1, which might be the critical inducers for OV progression. Notably, elevated CAFs and Macrophages_M2 infiltration could promote TGF-β, resulting in EMT activation.

We also noted that high-risk patients with Stage III-IV and Grade G3 exhibited dismal prognosis, high CAFs, increased TAM.M2 and a positive correlation with EMT-related pathways, which all contributed to the progression of OV and unfavorable prognosis to high-risk patients. However, some pathways such as natural killer cell-mediated cytotoxicity and mismatch repair, homologous recombination, antigen processing and presentation were apparently activated in low-risk patients, inducing more cytotoxicity on OV cells and provoking DNA repair in the OV tumor. Meanwhile, Plasma_cells and T_cells_CD8 were significantly accumulated in low-risk patients. Plasma cells differentiated from tumor infiltrating B cells or in tumor-draining lymph nodes are key mediators in anti-tumor immunity [50]. Meanwhile, CD8+ tumor-infiltrating cells can also exert inhibitory effects on the immunity in tumor and provide positive effects on the prognosis in virtually all solid tumors [51]. It is believed that accumulation of Plasma_cells and T_cells_CD8 could improve the survival of low-risk patients.

Next, 8 ferroptosis-related genes were used to develop a risk model using the TCGA-OV cohort to estimate the prognosis of OV patients, and its robustness in the GSE102073 cohort and ICGC-AU cohort was validated. The risk score showed positive correlation with PVR, CD276, and CD28 and negative correlation with LAG3, TIGIT, CTLA4, IDO1, CD27, ICOS, and IL2RB. Moreover, low-risk patients responded more actively to Cisplatin, Gemcitabine, and Gefitinib. Our findings also provide a direction for clinical strategies of chemotherapy in OV patients.

Some limitations in this study should be equally noted. Firstly, this study used retrospective data from public databases, therefore prospective study with large samples was required in the future. Secondly, the underlying mechanism of these ferroptosis-related genes in high-grade serous OV should be validated by *in vitro* and *in vivo* experimental studies. Finally, the reliability and long-term clinical application of the current model needed to be clinically verified.

## CONCLUSIONS

In summary, we developed and validated a novel prognostic risk model and molecular subtypes based on ferroptosis for high-grade serous OV, providing a direction to better understand the role of ferroptosis in OV. The current findings could facilitate the prognosis prediction and guide targeted therapies for treating high-grade serous OV patients.

## Supplementary Materials

Supplementary Figures
