# Supplementary material for: Defining three ferroptosis-based molecular subtypes and developing a prognostic risk model for high-grade serous ovarian cancer
Source: Aging (Albany NY). 2024 May 24;16(10):9106–26. doi: 10.18632/aging.205857 (PMC11164503; doi:10.18632/aging.205857)
Supplement: Supplementary Figures [file aging-16-205857-s001.pdf]

SUPPLEMENTARY FIGURE

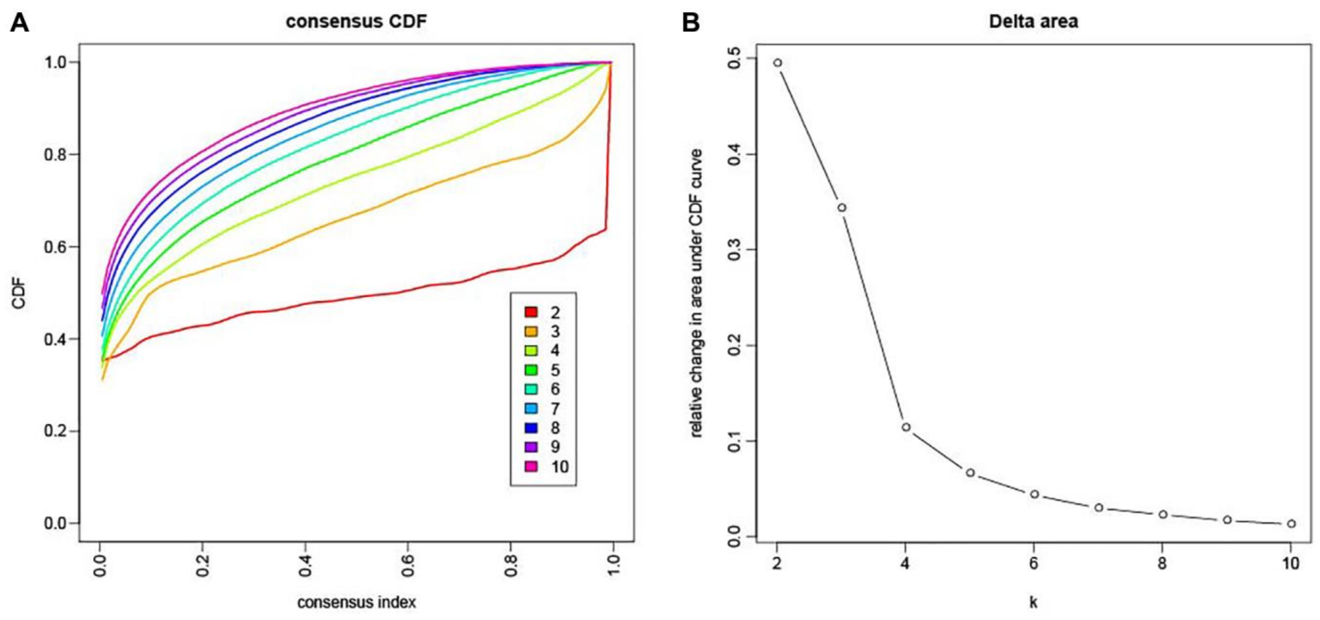

Supplementary Figure 1. Consensus clustering analysis in TCGA-OV cohort to identify optimal clusters. (**A**) Consensus CDF in TCGA cohort. (**B**) Relative changes in area under CDF Delta curve.
